# Effects of Structured Functional Turning Practice Within a Time-Matched Exercise Program on Functional Mobility, Gait Speed, and Balance in Early-Stage Parkinson’s Disease: A Randomized Controlled Trial

**DOI:** 10.3390/jcm15208044

**Published:** 2026-10-16

**Authors:** Kübra Uzun, Burak Buğday, Suzan Aydın, Ömer Şevgin

**Affiliations:** 1 Department of Physiotherapy and Rehabilitation, Institute of Health Sciences, Üsküdar University, Istanbul 34768, Türkiye; kubra.uzun3@st.uskudar.edu.tr; 2 Department of Gerontology, İnönü University, Malatya 44280, Türkiye; burak.bugday@inonu.edu.tr; 3 Department of Physiotherapy and Rehabilitation, Faculty of Health Sciences, Istanbul Gelişim University, Istanbul 34310, Türkiye; suaydin@gelisim.edu.tr; 4 Department of Physiotherapy and Rehabilitation, Faculty of Health Sciences, Üsküdar University, Istanbul 34768, Türkiye

**Keywords:** Parkinson’s disease, turning, exercise therapy, functional mobility, postural balance, gait

## Abstract

**Background/Objectives:** Turning frequently precipitates falls in Parkinson’s disease (PD) but is rarely trained systematically. We examined whether replacing part of a conventional exercise session with structured turning practice improves functional mobility, gait speed, and balance in early-stage PD. **Methods:** Fifty patients with PD (Hoehn and Yahr stage 1–2; aged 50–70 years) were randomized to a time-matched program in which 18–20 min of each 45–50 min session was devoted to turning practice and the remainder to a reduced conventional program (*n* = 25), or to a full conventional program (*n* = 25), three times weekly for 12 weeks. The primary outcome was the Timed Up and Go test (TUG); secondary outcomes were gait speed (10-Meter Walk Test) and the Berg Balance Scale (BBS). Outcome assessment was not blinded. Groups were compared with baseline-adjusted analysis of covariance (ANCOVA). **Results:** All participants completed the study. Adjusted differences favored the turning group for TUG (−1.79 s; 95% confidence interval [CI] −1.98 to −1.61), gait speed (0.116 m/s; 95% CI 0.108 to 0.124), and BBS (1.84 points; 95% CI 1.50 to 2.17) (all *p* < 0.001). **Conclusions:** In high-functioning patients with early-stage PD, a time-matched program including structured turning practice produced larger short-term improvements in TUG performance, gait speed, and balance than conventional exercise alone. The effects were unusually large and were obtained with unblinded assessment and with test tasks partly practiced during training; falls and turning quality were not measured. Assessor-blinded trials with turning-specific outcomes and fall recording are needed. Trial registration: ClinicalTrials.gov, NCT07442370.

## 1. Introduction

Bradykinesia, rigidity, tremor, and postural instability are the cardinal motor features of Parkinson’s disease (PD), a progressive neurodegenerative condition. Its global burden is rising quickly, and modelling based on the Global Burden of Disease data estimates that 25.2 million people will have PD in 2050, mostly because populations are ageing [1]. Falls are among the most disabling consequences of the disease. Pooling 54 studies, a meta-analysis found that almost half of people with PD (48%) reported at least one fall and about one third (32%) reported two or more [2]. Previous falls, gait disorders, and poor balance are the most consistent prognostic factors for future falls [3], which makes balance and gait natural targets for rehabilitation.

Turning is part of most daily activities and is particularly demanding for people with PD. Relative to age-matched adults without PD, they need more and shorter steps to turn and rotate the head later and more slowly [4], and the coordination between head and pelvis is altered during full 360° turns [5]. Turning time and the number of turning steps increase with disease stage and are related to postural instability [6], and turning characteristics are associated with the severity of freezing of gait [7]. Turning also carries prognostic information: gait and turning measures recorded with wearable sensors in daily life improved the prediction of future falls beyond fall history alone [8]. These problems are not confined to advanced disease. Many patients show abnormal balance and gait at the time of diagnosis, before any treatment is started [9], and reduced turning speed can be measured several years before PD is diagnosed clinically [10]. Turning is therefore a reasonable treatment target for exercise from the first years of the disease.

Physiotherapy and structured exercise form part of standard PD care. In a Cochrane network meta-analysis including 154 randomized trials, most forms of exercise reduced the severity of motor signs [11], and other network meta-analyses report benefits for balance, postural stability, general mobility, and gait [12,13,14]. Exercise probably lowers the fall rate in PD by roughly a quarter according to a Cochrane review [15], and balance, gait, and task-specific training carry strong recommendations in the guideline issued by the American Physical Therapy Association [16]. Conventional programs usually combine stretching, strengthening, balance, and gait exercises performed predominantly in straight-line or static conditions, and turning is seldom trained in a structured and progressive way. General gait training may not be sufficient to change turning: in a pilot study, overground locomotor training did not alter thoracic rotation during turns [17].

A small number of trials have targeted turning directly. In one trial, both a specific exercise program and treadmill training with built-in turning led to better turning in people with PD [18], and in another, task-specific movement practice reduced en bloc turning [19]. In a three-arm trial, a home-based exercise program delivered together with real or sham repetitive transcranial magnetic stimulation improved 180° turning characteristics compared with a control group [20]. A larger trial of a dedicated turning intervention has been designed, but only its protocol has been published [21]. These studies suggest that turning is trainable. However, they involved small samples, short programs of four to eight weeks, and specialized equipment or laboratory-based kinematic outcomes. Evidence on functional turning practice that can be delivered within routine physiotherapy sessions, and on its effect on standard clinical measures of mobility, gait, and balance, remains limited.

We therefore tested whether structured functional turning practice, introduced in place of part of a conventional exercise session so that total session time stayed the same, would improve functional mobility, gait speed, and balance in early-stage PD. Our hypothesis was that, compared with the conventional program, the turning program would shorten Timed Up and Go test (TUG) time to a greater extent, and that it would also lead to larger gains in gait speed and balance.

## 2. Materials and Methods

### 2.1. Study Design, Ethics, and Registration

We conducted a two-arm, parallel randomized controlled trial (allocation 1:1) at a single site, NRL Medical Center in Sancaktepe, Istanbul, Türkiye, from March to August 2026, and report it following the Consolidated Standards of Reporting Trials (CONSORT) 2025 statement [22]. Ethical approval was granted on 25 February 2026 by the Üsküdar University Non-Interventional Clinical Research Ethics Committee (meeting 02; file February 2026-28; reference 61351342/020-1919), the trial followed the principles of the Declaration of Helsinki, and every participant signed an informed consent form before enrollment. Patients and the public were not involved in the design, conduct, or reporting of the study.

The trial was registered at ClinicalTrials.gov (NCT07442370) on 25 February 2026, before enrollment of the first participant. The initial registry entry contained data-entry errors that did not correspond to the protocol approved by the ethics committee: it described a 6-week intervention with assessment at week 10, a virtual-reality adaptation of the TUG, masking of the care provider and outcome assessor, the Berg Balance Scale as a co-primary outcome, and turning exercises added to the full conventional program. The registry record was corrected after study completion, in October 2026, and its update history remains publicly available. Supplementary Table S1 compares the initial registry entry, the approved protocol, and the study as conducted. The approved protocol specified the sample size calculation and a between-group comparison with the independent-samples *t*-test; a separate statistical analysis plan was not prepared.

### 2.2. Eligibility Criteria

Participants were identified among patients attending the center’s physiotherapy and rehabilitation clinic. To be eligible, patients needed (i) idiopathic PD diagnosed clinically by a neurologist [23,24]; (ii) Hoehn and Yahr stage 1–2 [25]; (iii) age 50–70 years; (iv) a Mini-Mental State Examination score of 24 or higher; (v) stable medical treatment during the previous 6 weeks; (vi) stable vital signs; and (vii) to agree to take part and sign a consent form after receiving an explanation of the study. Exclusion criteria were (i) advanced PD (Hoehn and Yahr stage 3–5); (ii) serious orthopedic problems; (iii) vestibular disorders; and (iv) a fall accompanied by loss of consciousness within the previous 6 months. This last criterion was defined by the investigators for safety reasons, to exclude patients with possible syncopal events; it is not a standard definition of a fall, and patients with recurrent non-syncopal falls were not excluded.

### 2.3. Sample Size

The approved protocol based the target sample on a G*Power (version 3.1; Heinrich Heine University Düsseldorf, Düsseldorf, Germany) calculation for an independent-samples *t*-test between two groups, assuming a large effect size (d = 0.90), a significance level of α = 0.05, and a power of 0.90. The assumed effect was based on the large effects reported for turning performance after turning training in PD (η^2^ = 0.19–0.23) [18]; these correspond to Cohen’s f of 0.48–0.55 (f = √[η^2^/(1 − η^2^)]) and, for two groups, to d ≈ 2f = 0.97–1.09, so that d = 0.90 was a slightly more conservative assumption. In the original submission, the calculation was reported as one-sided. With a two-sided test, the same assumptions require 27 participants per group, and the 25 participants per group enrolled provide a power of approximately 0.88. No attrition allowance was planned, and no stopping rule was defined.

### 2.4. Randomization, Allocation Concealment, and Blinding

Allocation to the two groups (25 each) was made by drawing lots. Before the first patient was enrolled, the corresponding author (Ö.Ş.), who had no role in recruitment, treatment, or testing, prepared 50 identical slips of paper of the same size and thickness, marked 25 for each group, folded them in the same way, and placed them in an opaque bag, which was kept by the center’s secretary. The secretary likewise took no part in recruiting, treating, or testing participants. After a participant had given written consent, the secretary mixed the slips, drew one slip without replacement, and recorded the allocation in a written allocation log with the date and participant number. Allocation took place before the baseline assessment, but the result was kept by the secretary and was communicated to the treating physiotherapists only after the baseline assessment had been completed, so the assessor was unaware of group allocation at baseline. Because a fixed set of 50 slips was used, allocation was a restricted (balanced) randomization, and the remaining allocations became more predictable toward the end of recruitment.

Blinding of participants and physiotherapists was not feasible, given the type of intervention. All outcome assessments, at baseline and at week 12, were performed by one of the treating physiotherapists (K.U.), who was aware of group allocation at the week-12 assessment; the approved protocol did not specify assessor blinding. The trial is therefore open-label with unblinded outcome assessment. No video or automated recordings that would allow independent blinded reassessment are available.

### 2.5. Interventions

Both groups attended individual supervised sessions three times per week for 12 weeks (36 sessions). Total session duration was approximately 45–50 min in both groups. Sessions were delivered by four physiotherapists, each with at least 3 years of clinical experience, who were trained in both protocols before the study; each physiotherapist treated participants in both groups. Recruitment was staggered between March and May 2026; at most 21–30 participants were in the intervention period at the same time, and each physiotherapist delivered on average 3–4 study sessions per working day. The trial therefore compared two time-matched programs with different content, rather than an additional dose of exercise. Therapist contact time was matched by session duration, and both groups received the same individual supervision. The interventions are described according to the Template for Intervention Description and Replication (TIDieR) checklist (Supplementary Table S2).

Control group (conventional exercise). Participants received a conventional exercise program consisting of stretching, strengthening, balance, and gait exercises, which filled the whole session (Table 1). Exercise difficulty was progressed according to individual performance by increasing repetitions, reducing upper-extremity support, narrowing the base of support, increasing reaching distance, or increasing the complexity of gait tasks. The resistance of the dumbbells used in the strengthening exercises was increased every two weeks.

Experimental group (time-matched program with structured turning practice). Participants performed the same conventional exercises at a reduced dose and spent the remaining 18–20 min of each session on structured functional turning practice (Table 2). No conventional component was omitted: selected core exercises were performed with two sets and the remaining exercises with one set, and the balance and gait tasks were retained with fewer repetitions and a shorter duration. The turning program aimed to improve trunk rotation, directional changes, dynamic balance, and rotational control during functional activities, and progressed from seated rotational tasks to standing, walking, and balance-integrated tasks.

The program began with functional trunk rotation in sitting, in which participants reached toward targets on both sides while holding a ball (3 sets of 10 repetitions per side), followed by resisted trunk rotation, in which they moved an exercise ball toward lateral targets against an elastic band (Thera-Band; Performance Health, Akron, OH, USA; 2 sets of 10 repetitions per direction). Standing rotational tasks consisted of transferring an object from in front of the body to a target behind it, toward both sides (3 sets of 10 repetitions). Participants then walked three laps of a figure-of-eight path around two points approximately 2 m apart. Finally, rotation was combined with balance tasks: gentle trunk rotation in single-leg stance (3 × 30 s), trunk rotation while holding a ball in tandem stance (3 × 30 s), and a functional task in which participants stood up from a chair, turned 90° on verbal command, returned, and sat down, in both directions (3 sets of 10 repetitions). Elastic band resistance was increased every two weeks from red to green, blue, and black; the black band was reached in week 7 and maintained without further progression during weeks 9–12. Rest between sets was allowed as needed.

Safety, adherence, and fidelity. All standing balance tasks were performed under close supervision and guarding by the physiotherapist, and a task was stopped if the participant lost balance or reported pain or dizziness. Attendance was recorded at each session; the minutes delivered for each program component were not recorded. A session that could not be held on its scheduled day was rescheduled within the same week, so that three sessions per week were maintained. Adverse events during sessions, including falls, were monitored and recorded; falls and other events outside the sessions were not systematically recorded. Participants were asked not to start any other structured exercise program during the study; changes in antiparkinsonian medication were not recorded.

### 2.6. Outcome Measures

The primary outcome was the TUG; the secondary outcomes were gait speed in the 10-Meter Walk Test (10 MWT) and the Berg Balance Scale (BBS), as specified in the approved protocol. Participants were tested before the program and two days after their final (36th) session, at a similar time of day and in the “on” medication state, approximately 2–3 h after the morning dose of antiparkinsonian medication; the exact time of the last dose was not recorded. Tests were performed in a fixed order (BBS, TUG, 10 MWT) with a rest of 2–3 min between trials. Every test was repeated three times and the average value was used; for the BBS, the mean of the three total scores was rounded to the nearest whole number. Participants wore their usual footwear, and none used an assistive device.

Timed Up and Go test (TUG). From a seated position in a chair with armrests (seat height about 45 cm), participants rose, walked at a safe, comfortable speed to a floor marker 3 m away, turned around it in the direction of their choice, returned, and sat down again. Time was recorded with a handheld stopwatch to 0.01 s from the command “go” until the participant was seated again. The TUG measures functional mobility; a cut-off of 11.5 s has been reported to discriminate people with PD with and without a history of falls [26], but it does not measure fall risk directly. The test has high test–retest reliability in PD [27].

10-Meter Walk Test (10 MWT). Participants walked a 14 m path at their usual, comfortable speed. Only the middle 10 m was timed with a handheld stopwatch, leaving 2 m at each end for acceleration and deceleration, and gait speed (m/s) was derived from this time. Its test–retest reliability in PD is high [27].

Berg Balance Scale (BBS). The BBS rates 14 functional balance tasks on a 0–4 scale, giving a total score of up to 56, where a higher score reflects better balance. It is responsive to change after balance training in PD [28].

### 2.7. Statistical Analysis

Analyses were run in IBM SPSS Statistics for Windows, version 22 (IBM Corp., Armonk, NY, USA). We summarize continuous data as mean ± standard deviation (change scores additionally as median, interquartile range, and range); categorical variables are presented as number and percentage. Baseline characteristics are reported descriptively and were not tested for statistical significance.

Following the reviewers’ recommendations, the primary analysis of each outcome was an analysis of covariance (ANCOVA) of the week-12 value, entering group as a factor and adjusting for the participant’s baseline score on that outcome, the approach recommended when outcomes are measured before and after randomization [29]. Each model was also fitted with the change score (week-12 value minus baseline) as the dependent variable, group as the independent variable, and the baseline score as the covariate; this model estimates the baseline-adjusted between-group difference in improvement and yields the same group estimate, 95% confidence interval (CI), F statistic, and *p* value as the model for the week-12 value. Results are reported as adjusted group means and the adjusted between-group difference with its 95% CI. Model assumptions were checked before the group effect was interpreted: homogeneity of regression slopes by adding a group-by-baseline interaction term to each model, normality of residuals with the Shapiro–Wilk test, homogeneity of variances with Levene’s test, linearity by inspection of scatterplots of baseline against week-12 values in each group, and influential observations with Cook’s distance. Effect sizes were reported as partial eta squared (ηp^2^ = F/[F + residual degrees of freedom]) and as a standardized difference d, calculated as the adjusted mean difference divided by the pooled baseline standard deviation. The TUG was the primary outcome and was tested at α = 0.05 (two-sided); for the two secondary outcomes, a Bonferroni-adjusted threshold of α = 0.025 was applied.

The analysis specified in the protocol, the independent-samples *t*-test, is reported as a sensitivity analysis applied to change scores. Further post hoc sensitivity analyses included ANCOVA additionally adjusted for body mass index, a rank-based ANCOVA, and the Mann–Whitney U test for change scores. As a post hoc responder analysis, the proportion of participants in each group whose individual change reached a published threshold was compared with the risk difference and its Newcombe 95% CI: an improvement of at least 1.27 s for the TUG (upper bound of the minimal clinically important difference [30]), at least 0.082 m/s for gait speed [31], and at least 2 points for the BBS, rounding up its reported minimal important difference of 1.97 points [28]. No interim analyses were performed. All randomized participants completed the study, there were no missing data, and all analyses included all 50 participants in the groups to which they were randomized, so that the intention-to-treat and per-protocol populations were identical.

## 3. Results

### 3.1. Participants

**Figure 1:**
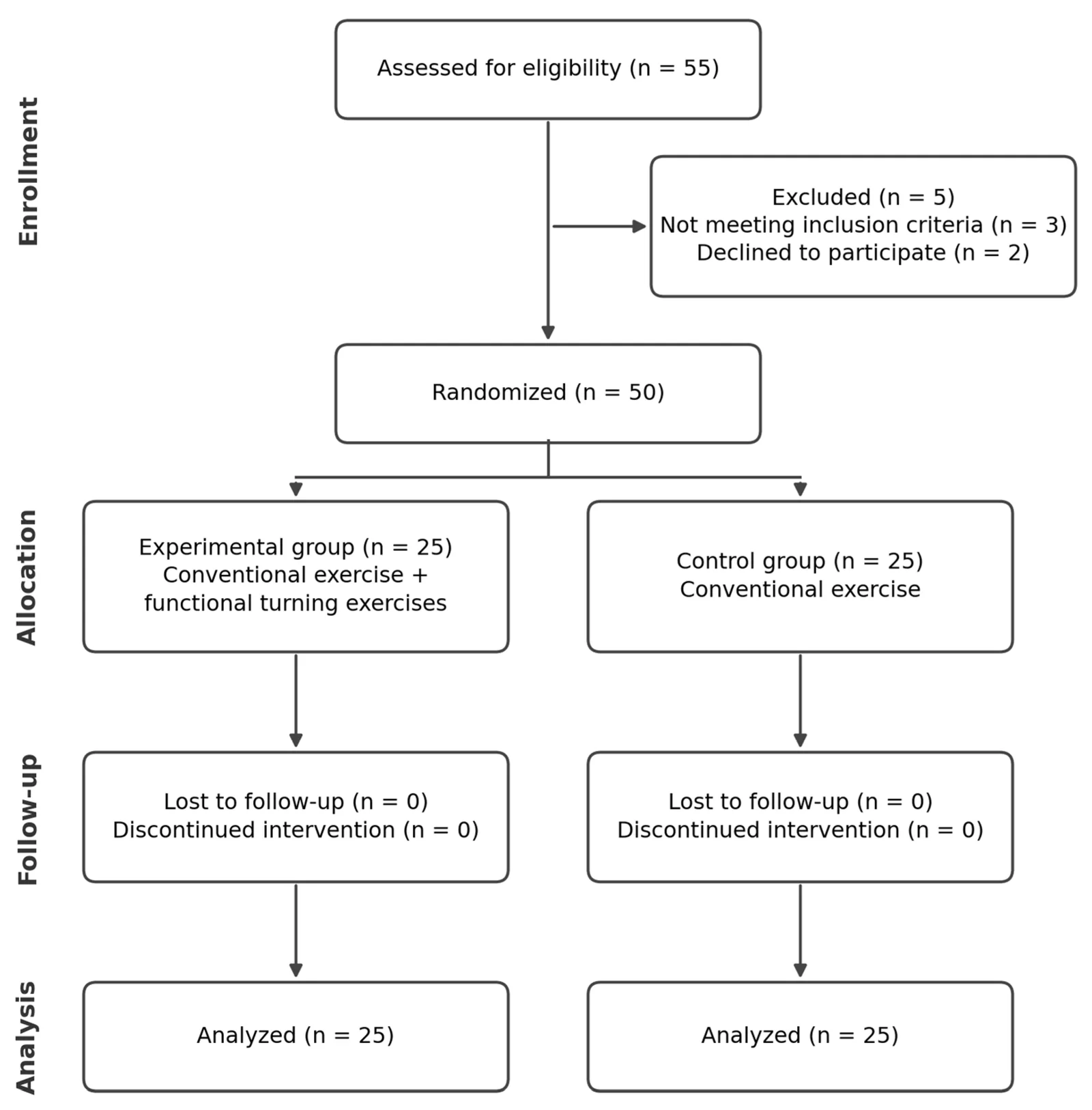
Flow of participants through the trial (Consolidated Standards of Reporting Trials [CONSORT] diagram).

Of 55 patients screened, three did not meet the eligibility criteria (two had Hoehn and Yahr stage 3 or higher and one had an orthopedic or vestibular disorder) and two declined to participate (Figure 1). The remaining 50 were randomized (25 per group). According to the attendance records, every participant attended all 36 sessions and completed the week-12 tests, and all 50 were analyzed. No falls or other adverse events were recorded during the sessions in either group.

Baseline characteristics are shown in Table 3. The groups were similar in age, sex, and anthropometric characteristics. Baseline BBS scores were higher in the experimental group (50.20 ± 1.22 vs. 48.60 ± 1.41 points), and six participants in each group had a baseline TUG time above 11.5 s. Disease duration, the distribution of Hoehn and Yahr stages 1 and 2, freezing of gait, previous falls, and antiparkinsonian medication were not recorded, so baseline comparability on these prognostic factors cannot be established.

### 3.2. Primary and Secondary Outcomes

**Figure 2:**
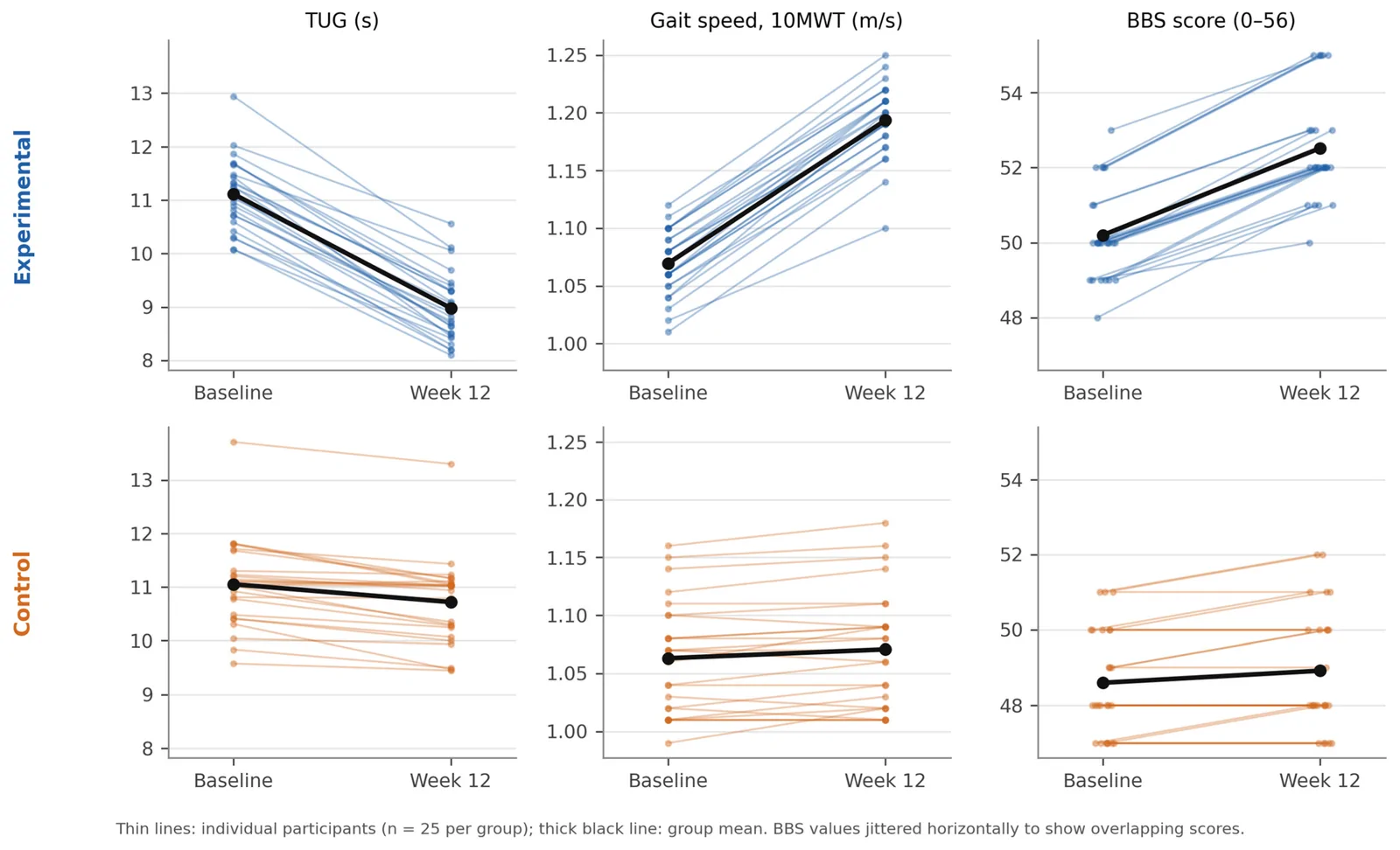
Individual participant values at baseline and week 12 in the experimental (**top**) and control (**bottom**) groups for the Timed Up and Go test, gait speed in the 10-Meter Walk Test, and the Berg Balance Scale (BBS). Thin lines show individual participants and the thick black line the group mean; BBS values are jittered horizontally to show overlapping scores.

Outcome values and the baseline-adjusted between-group differences are shown in Table 4, and individual participant trajectories in Figure 2. For the primary outcome, participants in the experimental group completed the TUG 1.79 s faster at week 12 after baseline adjustment (95% CI 1.61 to 1.98; *p* < 0.001). For the secondary outcomes, adjusted gait speed was 0.116 m/s higher (95% CI 0.108 to 0.124; *p* < 0.001) and the adjusted BBS score was 1.84 points higher (95% CI 1.50 to 2.17; *p* < 0.001) in the experimental group. The ANCOVA of change scores (Table 5) showed that, after adjustment for baseline, the TUG improved by a mean of 2.13 s in the experimental group and 0.34 s in the control group, gait speed by 0.124 and 0.008 m/s, and the BBS by 2.24 and 0.40 points, respectively; the adjusted between-group differences in improvement were identical to those in Table 4. The group-by-baseline interaction was not significant for any outcome (TUG F(1, 46) = 1.00, *p* = 0.32; gait speed F(1, 46) = 0.01, *p* = 0.91; BBS F(1, 46) = 0.02, *p* = 0.90), so the assumption of homogeneous regression slopes was met and the interaction term was not retained. Variances were similar between groups (Levene *p* ≥ 0.37), and no observation was unduly influential (maximum Cook’s distance 0.27). Residuals were normally distributed for the TUG and gait speed (Shapiro–Wilk *p* = 0.60 and 0.18) but not for the BBS (*p* < 0.001), reflecting its discrete scale; the rank-based ANCOVA (Section 3.4) gave the same conclusion.

### 3.3. Responder Analysis

In the post hoc responder analysis, all 25 participants in the experimental group and none in the control group improved by at least 1.27 s in the TUG (risk difference 1.00; 95% CI 0.81 to 1.00). Twenty-four participants in the experimental group and none in the control group improved by at least 0.082 m/s in gait speed and by at least 2 points in the BBS (risk difference 0.96; 95% CI 0.76 to 0.99 for both). Change-score distributions in the two groups did not overlap for any outcome (Table 4, Figure 2).

### 3.4. Sensitivity Analyses

The protocol-specified comparison of change scores gave between-group differences of −1.80 s (95% CI −1.99 to −1.61) for the TUG, 0.116 m/s (0.109 to 0.124) for gait speed, and 2.00 points (1.71 to 2.29) for the BBS. Additional adjustment for body mass index (−1.80 s, 0.118 m/s, and 1.89 points), rank-based ANCOVA, and Mann–Whitney U tests of change scores gave the same conclusions (all *p* < 0.001).

## 4. Discussion

In this randomized trial in high-functioning patients with early-stage PD, a 12-week, time-matched exercise program in which 18–20 min of each session was devoted to structured functional turning practice produced larger short-term improvements in TUG performance, gait speed, and BBS score than a conventional program of the same duration. Because the experimental group performed a reduced conventional program, the comparison is between two programs of different content rather than between an intervention and an additional dose of exercise, and the effects cannot be attributed to turning practice alone.

The largest difference was observed in the primary outcome, the TUG. This finding should be interpreted with caution. The TUG consists of rising from a chair, walking, turning 180°, and sitting down, and the experimental program included repeated sit-to-stand, turning, and return-to-chair tasks that closely resemble the test. Part of the improvement may therefore reflect near transfer or familiarity with the test task rather than a general improvement in mobility. The program also included single-leg and tandem stance, weight shifting, and figure-of-eight walking, so its effects may result from the combination of balance, rotational, and task-specific components. People with PD turn with more and smaller steps and with altered head and pelvis rotation [4,5], and turning-based treadmill training, a task-specific movement program, and home-based exercise have improved turning in previous trials [18,19,20], whereas general overground locomotor training did not change trunk rotation during turns [17]. Because no turning-specific outcome was measured in the present study, however, our results do not show that turning quality itself changed.

The study did not measure falls. Baseline TUG times in both groups (approximately 11 s) were close to the 11.5 s value that discriminated people with PD with and without a history of falls [26], and the mean TUG time of the experimental group was well below this value at week 12. This cut-off was derived from retrospective fall history and does not allow conclusions about future fall rates; moreover, although exercise probably reduces the rate of falls in PD [15], which types of exercise prevent falls, and in whom, remains uncertain [32]. Our findings should therefore be read as an effect on functional mobility, gait speed, and balance performance, not as evidence of fall prevention.

The gains in gait speed and BBS score are consistent with network meta-analyses showing that exercise improves balance, mobility, and gait in PD [11,12,13,14], with a meta-analysis showing that specific trunk exercises improve BBS scores [33], a dose–response meta-analysis reporting a moderate effect of physiotherapy on balance [34], and trials of dance, resistance, and balance training [35,36]. Other approaches based on motor learning and structured functional movement, such as action observation and motor imagery [37] and Argentine tango [38], have also improved balance and mobility in PD. Like the present program, these interventions practice complex whole-body movements, but they differ from it in their mechanisms (observation and mental rehearsal of movement in the former, rhythmic partnered dancing in the latter), and their effects cannot be transferred directly to the program tested here. Part of the reason for the small gains in the control group may be that participants were already functioning well: all had mild disease (Hoehn and Yahr 1 or 2), and with BBS scores of 47 to 53 of a possible 56 at baseline, the scale could register only small further gains.

Because the groups differed in baseline BBS scores by chance (50.20 vs. 48.60 points), inference was based on baseline-adjusted estimates, and the assumptions of these models, including homogeneous regression slopes, were met. Adjustment mattered mainly for the BBS: the adjusted difference in improvement (1.84 points) was smaller than the unadjusted difference in change scores (2.00 points), because the experimental group started higher and, within groups, higher baseline scores were associated with slightly larger gains, whereas the adjusted and unadjusted estimates for the TUG and gait speed were almost identical. Baseline imbalance therefore does not explain the direction of the findings, but it slightly inflates the unadjusted BBS effect.

The clinical importance of the findings depends on the threshold used, and thresholds of different types should not be confused. Minimal detectable change values indicate measurement error, whereas minimal clinically important differences indicate change that patients perceive as meaningful; published values also differ by disease severity and measurement method. For the TUG, the adjusted between-group difference of 1.79 s exceeded the reported minimal clinically important difference of 0.71–1.27 s but not the 2.04 s threshold for substantial clinical benefit [30], and it was below the earlier minimal detectable change of 3.5 s [39]; the TUG has also shown substantial responsiveness in PD [40]. For the BBS, the adjusted difference of 1.84 points was below the minimal important difference of 1.97 points [28] and the earlier minimal detectable change of 5 points [41]. For gait speed, the difference of 0.116 m/s exceeded the minimal clinically important difference of 0.082 m/s [31], but that value was derived from instrumented gait analysis and may not apply directly to a stopwatch-timed 10 MWT. The between-group differences were therefore statistically clear, but their clinical importance is uncertain, and the durability of the effects is unknown.

The size and consistency of the effects were unusual for an exercise trial in PD. Standardized differences ranged from 1.4 to 2.9, all participants in the experimental group and none in the control group reached the TUG threshold, and the distributions of change scores did not overlap. These results arise mainly from the very small variability of the change scores. Possible explanations include the homogeneous, high-functioning sample, individual supervision, averaging of three trials per test, the close similarity between the training and test tasks, and, importantly, unblinded outcome assessment at week 12 by a treating physiotherapist who knew each participant’s allocation. Detection bias of this kind could inflate the effects, particularly for the observer-rated BBS and for stopwatch timing. To allow independent verification, de-identified participant-level data are provided as Supplementary Data S3. These findings should be confirmed in assessor-blinded trials before they are used to guide practice.

### Limitations

Several limitations should be considered. First, all outcome assessments were performed by one of the treating physiotherapists, who was aware of group allocation at the week-12 assessment, and neither participants nor physiotherapists were blinded; this introduces a risk of performance and detection bias, particularly for the BBS. In addition, the four physiotherapists treated participants in both groups, so some contamination of the control program with turning elements cannot be excluded. Second, the initial registry entry did not match the approved protocol, and the record was corrected only after study completion; although the study followed the approved protocol (Supplementary Table S1), readers should consider this discrepancy when judging the trial. Third, the primary analysis (ANCOVA) differs from the *t*-test specified in the protocol, and the responder and other sensitivity analyses were post hoc, although all analyses gave consistent results. Fourth, no turning-specific outcome (for example, turn duration, number of turning steps, or a 360° turn test) was included, the primary outcome includes tasks that were practiced during training, and falls were not recorded; the proposed turning-related mechanism and any effect on falls therefore remain untested. Fifth, the experimental program was multicomponent and replaced part of the conventional program, so its effects cannot be attributed specifically to turning practice. Sixth, disease duration, the distribution of Hoehn and Yahr stages, motor severity, freezing of gait, fall history, and antiparkinsonian medication were not recorded, so prognostic imbalance between the groups cannot be excluded, the timing of assessments relative to the last medication dose was not standardized, and each test, including the full BBS, was administered three times at each assessment, which may have introduced practice and fatigue effects. Only attendance was recorded at each session, not the minutes delivered for each program component, so fidelity to the planned dose could not be quantified. Seventh, all participants came from one center, were between 50 and 70 years of age, and were in Hoehn and Yahr stages 1 or 2, with high baseline BBS scores and a possible ceiling effect, which limits generalization to older patients and to those with more advanced disease or freezing of gait. Finally, there was no follow-up after the intervention, so the durability of the effects is unknown. Future trials should use blinded assessors, record falls prospectively, include turning-specific outcomes not practiced during training, and consider wearable inertial sensors, which allow objective, multidimensional assessment of gait and turning in PD [8,42].

## 5. Conclusions

In high-functioning patients with early-stage Parkinson’s disease, a 12-week, time-matched exercise program that replaced part of conventional exercise with structured functional turning practice led to greater short-term gains than conventional exercise alone in TUG time, walking speed, and balance. The effects were unusually large and were obtained with unblinded outcome assessment and with a primary outcome whose components were practiced during training; falls and turning quality were not measured, and the clinical importance of the between-group differences depends on the threshold used. The program can be delivered with low-cost, commonly available equipment, but its effects should be confirmed in assessor-blinded trials with turning-specific outcomes, prospective fall recording, and follow-up.

## Figures and Tables

**Table 1:** Conventional exercise program (control group, full dose).

Component	Exercises	Dose	Approximate Duration
Stretching	Pectoral muscles, trunk rotators, hip flexors, hamstrings, quadriceps, and gastrocnemius–soleus	2 sets × 20–30 s per muscle group	8–10 min
Strengthening	Sit-to-stand, mini-squat, standing hip abduction and extension, knee extension, heel raises, and trunk extensor exercises	2 sets × 10–12 repetitions per exercise; rest as required	10–12 min
Balance training	Anterior–posterior and lateral weight shifting, reaching activities, semi-tandem and tandem stance, and multidirectional stepping	Dynamic tasks: 2 sets × 8–10 repetitions; static tasks: 2 × 20–30 s	10–12 min
Gait training	Overground walking with emphasis on step length and arm swing, start–stop practice, obstacle crossing, and directional changes	2–3 walking bouts of 2–3 min; 5–8 task repetitions	10–12 min

**Table 2:** Functional turning practice (experimental group, 18–20 min per session).

Exercise	Starting Position	Application	Dose	Primary Target
Ball-reaching trunk rotation	Sitting	Active trunk rotation by reaching with a ball toward targets placed on the right and left sides	3 × 10 repetitions per direction	Trunk rotation; functional reaching
Resisted trunk rotation	Sitting	Moving an exercise ball toward right and left targets against the resistance of an elastic band	2 × 10 repetitions per direction	Rotational strength; trunk control
Object transfer with rotation	Standing	Picking up an object positioned in front and placing it behind the body by rotating the trunk	3 × 10 repetitions	Trunk rotation; weight shifting; functional reaching
Figure-of-eight walking	Standing/walking	Walking around two turning points about 2 m apart in a figure-of-eight trajectory	3 laps	Directional change; dynamic balance; turning control
Single-leg trunk rotation	Single-leg stance	Gentle trunk rotation while maintaining single-leg stance	3 × 30 s	Integration of balance and rotation
Ball rotation in tandem stance	Tandem stance	Trunk rotation to both sides while holding a ball in tandem stance	3 × 30 s	Postural control; rotation with reduced base of support
Functional 90° turning task	Sitting on a chair	Rising, turning 90° when cued, walking back, and sitting	3 × 10 repetitions	Functional turning; mobility; postural control

**Table 3:** Baseline characteristics of the participants.

Characteristic	Experimental Group (*n* = 25)	Control Group (*n* = 25)	Total (*n* = 50)
Age (years), mean ± SD	60.16 ± 4.06	60.68 ± 3.80	60.42 ± 3.90
Female sex, *n* (%)	13 (52.0)	12 (48.0)	25 (50.0)
Height (cm), mean ± SD	166.24 ± 8.33	167.68 ± 6.58	166.96 ± 7.47
Body weight (kg), mean ± SD	73.04 ± 9.85	71.00 ± 7.31	72.02 ± 8.65
Body mass index (kg/m^2^), mean ± SD	26.36 ± 2.34	25.20 ± 1.53	25.78 ± 2.04
Married, *n* (%)	24 (96.0)	23 (92.0)	47 (94.0)
Single or divorced/widowed, *n* (%)	1 (4.0)	2 (8.0)	3 (6.0)
Education: literate or primary/secondary school, *n* (%)	18 (72.0)	17 (68.0)	35 (70.0)
Education: high school or higher, *n* (%)	7 (28.0)	8 (32.0)	15 (30.0)
TUG (s), mean ± SD	11.11 ± 0.67	11.05 ± 0.82	11.08 ± 0.74
Gait speed, 10 MWT (m/s), mean ± SD	1.07 ± 0.03	1.06 ± 0.05	1.07 ± 0.04
BBS (points), mean ± SD	50.20 ± 1.22	48.60 ± 1.41	49.40 ± 1.54

Abbreviations: BBS, Berg Balance Scale; SD, standard deviation; TUG, Timed Up and Go test; 10 MWT, 10-Meter Walk Test.

**Table 4:** Outcome measures and baseline-adjusted between-group differences.

Outcome	Group	Baseline, Mean ± SD	Week 12, Mean ± SD	Change, Mean ± SD (Range)	Adjusted Mean at Week 12 (95% CI)	Adjusted Difference (95% CI)	*p*	ηp^2^; d
TUG (s)	Experimental	11.11 ± 0.67	8.98 ± 0.65	−2.13 ± 0.38 (−2.98 to −1.41)	8.95 (8.82 to 9.08)	−1.79 (−1.98 to −1.61)	<0.001	0.89; −2.39
	Control	11.05 ± 0.82	10.72 ± 0.81	−0.33 ± 0.27 (−0.84 to 0.07)	10.75 (10.62 to 10.88)			
Gait speed (m/s)	Experimental	1.07 ± 0.03	1.19 ± 0.03	0.124 ± 0.016 (0.08 to 0.16)	1.190 (1.185 to 1.196)	0.116 (0.108 to 0.124)	<0.001	0.95; 2.90
	Control	1.06 ± 0.05	1.07 ± 0.05	0.008 ± 0.012 (−0.01 to 0.03)	1.074 (1.068 to 1.080)			
BBS (points)	Experimental	50.20 ± 1.22	52.52 ± 1.45	2.32 ± 0.56 (1 to 3)	51.64 (51.42 to 51.86)	1.84 (1.50 to 2.17)	<0.001	0.72; 1.39
	Control	48.60 ± 1.41	48.92 ± 1.63	0.32 ± 0.48 (0 to 1)	49.80 (49.58 to 50.02)			

CI: confidence interval; BBS, Berg Balance Scale; SD, standard deviation; TUG, Timed Up and Go test. Adjusted estimates come from analysis of covariance (ANCOVA) models including group and the baseline score (F(1, 47) = 386.9, 854.0, and 119.5, respectively). ηp^2^: partial eta squared; d: adjusted difference divided by the pooled baseline standard deviation. Negative TUG values favor the experimental group.

**Table 5:** Analysis of covariance (ANCOVA) of change scores (week 12 minus baseline) adjusted for baseline, and model assumption checks.

Outcome	Adjusted Mean Change, Experimental (95% CI)	Adjusted Mean Change, Control (95% CI)	Adjusted Difference in Change (95% CI)	F(1, 47); *p*; ηp^2^	Group × Baseline Interaction, F(1, 46); *p*	Levene *p*; Shapiro–Wilk *p*
TUG (s)	−2.13 (−2.26 to −2.00)	−0.34 (−0.47 to −0.21)	−1.79 (−1.98 to −1.61)	386.9; <0.001; 0.89	1.00; 0.32	0.37; 0.60
Gait speed (m/s)	0.124 (0.118 to 0.130)	0.008 (0.002 to 0.013)	0.116 (0.108 to 0.124)	854.0; <0.001; 0.95	0.01; 0.91	0.54; 0.18
BBS (points)	2.24 (2.02 to 2.46)	0.40 (0.18 to 0.62)	1.84 (1.50 to 2.17)	119.5; <0.001; 0.72	0.02; 0.90	0.51; <0.001

CI: confidence interval; BBS, Berg Balance Scale; TUG, Timed Up and Go test; ηp^2^, partial eta squared. Models include group as the independent variable and the baseline score of the outcome as the covariate; adjusted means are evaluated at the overall baseline mean. The interaction term tests homogeneity of regression slopes and was not retained in the final models. Levene and Shapiro–Wilk tests refer to the model residuals.

## Data Availability

De-identified participant-level data are provided as Supplementary Data S3. Further information is available from the corresponding author on reasonable request.

## Supplementary Materials

The following supporting information can be downloaded at: https://www.mdpi.com/article/10.3390/jcm15208044/s1, Table S1: Comparison of the initial registry entry, the approved protocol, and the conducted study; Table S2: TIDieR checklist [43]; Data S3: De-identified participant-level data; CONSORT 2025 checklist [22].
