# Supplementary material for: Effects of Structured Functional Turning Practice Within a Time-Matched Exercise Program on Functional Mobility, Gait Speed, and Balance in Early-Stage Parkinson’s Disease: A Randomized Controlled Trial
Source: J Clin Med. 2026 Oct 16;15(20):8044. doi: 10.3390/jcm15208044 (PMC13706036; doi:10.3390/jcm15208044)
Supplement: Supplementary file 1 [file jcm-15-08044-s001.zip › CONSORT_2025_Checklist_NCT07442370.pdf]

CONSORT 2025 Checklist

Manuscript: *Effects of Functional Turning Exercises Added to Conventional Exercise on Fall Risk, Gait Speed, and Balance in Patients with Parkinson's Disease: A Randomized Controlled Trial*

Journal of Clinical Medicine · Corresponding author: Ömer Şevgin · Trial registration: ClinicalTrials.gov NCT07442370

Section/Topic	Item No	Checklist item (summary)	Reported in manuscript
**Title and abstract**			
Title	1a	Identification as a randomised trial	Title ("A Randomized Controlled Trial")
Abstract	1b	Structured summary of design, methods, results, conclusions, registration and funding	Abstract (incl. trial registration: ClinicalTrials.gov, NCT07442370, 25 February 2026); funding: Back matter, Funding statement
**Open science**			
Trial registration	2	Registry name, identifier and URL, date of registration	Abstract; Section 2.1 (ClinicalTrials.gov, NCT07442370, prospectively registered on 25 February 2026, before enrolment of the first participant)
Protocol and statistical analysis plan	3	Where the protocol and statistical analysis plan can be accessed	Section 2.1 (not published separately; registered trial record publicly available)
Data sharing	4	What data are shared, where and how	Back matter, Data Availability Statement (available from the corresponding author on reasonable request)
Funding and conflicts of interest	5a	Sources of funding and role of funders	Back matter, Funding statement (no external funding)
	5b	Financial and other conflicts of interest of the authors	Back matter, Conflicts of Interest (none declared)
**Introduction**			
Background and rationale	6	Scientific background and rationale	Section 1, paragraphs 1–4
Objectives	7	Specific objectives related to benefits and harms	Section 1, final paragraph (aim and hypothesis)
**Methods**			
Patient and public involvement	8	Details of patient or public involvement	Section 2.1 (patients and the public were not involved)
Trial design	9	Type of trial, framework, unit of randomisation, allocation ratio	Section 2.1 (single-centre, parallel-group RCT; individual randomisation; 1:1)
Changes to trial protocol	10	Important changes to the trial after it commenced, with reasons	None. The trial was conducted as registered; after completion, the ClinicalTrials.gov record was updated with the actual study dates, enrolment, and intervention details

Section/Topic	Item No	Checklist item (summary)	Reported in manuscript
Trial setting	11	Settings and locations where the trial was conducted	Section 2.1 (NRL Medical Center, Sancaktepe, Istanbul, Türkiye; March–August 2026); Section 2.2 (recruitment from the physiotherapy and rehabilitation clinic)
Eligibility criteria	12a	Eligibility criteria for participants	Section 2.2
	12b	Eligibility criteria for sites and individuals delivering the interventions	Section 2.5 (single physiotherapist with 3 years of clinical experience; individual sessions)
Intervention and comparator	13	Interventions with sufficient details to allow replication	Section 2.5; Table 1 (conventional program); Table 2 (functional turning program); dose, frequency, duration and progression
Outcomes	14	Pre-specified primary and secondary outcomes, how and when assessed	Section 2.6 (primary: TUG; secondary: 10MWT, BBS; baseline and 2 days after the 12-week intervention; mean of three trials)
Harms	15	How harms were defined and assessed	Section 2.5 (adverse events, including falls during sessions, monitored throughout the study)
Sample size	16a	How sample size was determined	Section 2.3 (G*Power 3.1; d = 0.90, α = 0.05, power 0.90; 22 per group; 25 per group enrolled)
Interim analyses and stopping guidelines	16b	Interim analyses and stopping guidelines	Section 2.7 (no interim analyses planned or performed)
Randomisation: sequence generation	17a	Who generated the random allocation sequence and the method used	Section 2.4 (drawing of lots by the centre secretary)
Randomisation: type	17b	Type of randomisation and any restriction	Section 2.4 (50 slips, 25 per group, drawn without replacement, giving equal group sizes; no stratification)
Allocation concealment mechanism	18	Mechanism used to conceal the sequence until assignment	Section 2.4 (identical folded slips in an opaque bag)
Implementation	19	Who enrolled participants and who assigned them to interventions	Section 2.4 (secretary not involved in recruitment, treatment or assessment drew one slip per participant in order of enrolment)
Blinding	20a	Who was blinded after assignment	Section 2.4 (participants, treating physiotherapist and outcome assessor not blinded); Section 4.1 (limitation)
	20b	How blinding was achieved and maintained	Not applicable (no blinding)
Statistical methods	21a	Statistical methods used to compare groups	Section 2.7
	21b	Definition of who is included in each analysis	Section 2.7 and Section 3.1 (all 50 randomised participants analysed in the groups to which they were allocated)
	21c	How missing data were handled	Section 2.7 (no missing data)

Section/Topic	Item No	Checklist item (summary)	Reported in manuscript
	21d	Any other analyses, distinguishing pre-specified from post hoc	Section 2.7 (ANCOVA and non-parametric analyses performed post hoc as sensitivity analyses)
**Results**			
Participant flow	22a	Numbers randomly assigned, receiving treatment and analysed, per group	Section 3.1; Figure 1 (CONSORT flow diagram)
Losses and exclusions	22b	Losses and exclusions after randomisation, with reasons	Section 3.1; Figure 1 (no losses or exclusions)
Recruitment	23a	Dates of recruitment and follow-up	Section 2.1 (March–August 2026); Section 2.6 (post-intervention assessment 2 days after week 12)
	23b	Why the trial ended or was stopped	Not applicable (completed as planned)
Intervention and comparator delivery	24a	Intervention delivery and adherence	Section 2.5; Section 3.1 (all participants completed all 36 sessions)
Concomitant care	24b	Concomitant care received by participants	Section 2.2 (stable medical treatment for 6 weeks before enrolment); antiparkinsonian medication not recorded, Section 4.1
Baseline data	25	Baseline demographic and clinical characteristics for each group	Section 3.1; Table 3
Numbers analysed, outcomes and estimation	26	Numbers analysed, results for each group and effect size with precision	Sections 3.2–3.4; Tables 4–6; Figures 2–3 (n = 25 per group; mean differences with 95% CI, effect sizes)
Harms	27	All important harms or unintended effects in each group	Section 3.1 (no adverse events in either group)
Ancillary analyses	28	Other analyses performed, distinguishing pre-specified from post hoc	Section 3.4 (baseline-adjusted and non-parametric analyses, post hoc)
**Discussion**			
Interpretation	29	Interpretation consistent with results, balancing benefits and harms	Section 4
Limitations	30	Limitations, sources of bias, imprecision, generalisability	Section 4.1

Item content is summarised from: Hopewell S, et al. CONSORT 2025 statement: updated guideline for reporting randomised trials. BMJ 2025;389:e081123. Locations refer to section, table and figure numbers of the submitted manuscript.
