# Supplementary material for: Effects of Structured Functional Turning Practice Within a Time-Matched Exercise Program on Functional Mobility, Gait Speed, and Balance in Early-Stage Parkinson’s Disease: A Randomized Controlled Trial
Source: J Clin Med. 2026 Oct 16;15(20):8044. doi: 10.3390/jcm15208044 (PMC13706036; doi:10.3390/jcm15208044)
Supplement: Supplementary file 1 [file jcm-15-08044-s001.zip › Table_S1_registry_protocol_comparison.pdf]

Supplementary Table S1. Comparison of the initial registry entry, the ethics-approved protocol, and the study as conducted

Trial: Effects of Structured Functional Turning Practice Within a Time-Matched Exercise Program on Functional Mobility, Gait Speed, and Balance in Early-Stage Parkinson's Disease. ClinicalTrials.gov NCT07442370; ethics approval 61351342/020-1919.

Item	Initial ClinicalTrials.gov entry (25 February 2026)	Ethics-approved protocol (25 February 2026)	Study as conducted (March–August 2026)	Explanation
Recruitment status and enrollment	Recruiting; estimated enrollment 50	Target sample size 50 (25 per group)	Completed; 50 randomized, 50 analyzed	Record not updated during the study; updated after completion
Intervention duration	6 weeks	12 weeks	12 weeks (36 sessions)	Data-entry error in the initial registry entry
Session frequency and duration	3 sessions/week; 40–50 min	3 sessions/week; 45–50 min	3 sessions/week; approximately 45–50 min	Data-entry error in the initial registry entry
Timing of post-intervention assessment	Week 10	After the 12-week intervention	2 days after the final session (week 12)	Data-entry error in the initial registry entry
Primary outcome(s)	TUG and BBS	TUG	TUG	Data-entry error in the initial registry entry
Secondary outcomes	10MWT	10MWT, BBS	10MWT (gait speed), BBS	
TUG procedure	Virtual-reality adaptation of the TUG	Standard TUG	Standard TUG (stopwatch)	Data-entry error in the initial registry entry
Masking	Care provider and outcome assessor	No blinding specified	None (open label); all outcomes assessed by one of the treating physiotherapists	Data-entry error in the initial registry entry
Experimental intervention	Turning exercises added to full conventional therapy	Time-matched: part of the conventional program replaced by turning exercises	Time-matched: reduced conventional program plus 18–20 min of turning practice	Data-entry error in the initial registry entry
Planned analysis	Not stated	Independent-samples t-test between groups; G*Power d = 0.90, α = 0.05, power 0.90	Primary: ANCOVA (baseline-adjusted); protocol t-test reported as sensitivity analysis	ANCOVA adopted at revision on reviewers' recommendation

TUG: Timed Up and Go test; BBS: Berg Balance Scale; 10MWT: 10-Meter Walk Test. The registry record was corrected in October 2026, after study completion; all versions remain available in the record's update history.
