# Supplementary material for: Effects of Structured Functional Turning Practice Within a Time-Matched Exercise Program on Functional Mobility, Gait Speed, and Balance in Early-Stage Parkinson’s Disease: A Randomized Controlled Trial
Source: J Clin Med. 2026 Oct 16;15(20):8044. doi: 10.3390/jcm15208044 (PMC13706036; doi:10.3390/jcm15208044)
Supplement: Supplementary file 1 [file jcm-15-08044-s001.zip › Table_S2_TIDieR.pdf]

Supplementary Table S2. TIDieR checklist

TIDieR item	Experimental group: time-matched program with turning practice	Control group: conventional exercise
1. Brief name	Structured functional turning practice within a time-matched exercise program	Conventional exercise program
2. Why	Turning is impaired in PD and is a common circumstance of falls, but is rarely trained systematically in conventional programs	Usual-care comparator reflecting guideline-recommended stretching, strengthening, balance, and gait training
3. What (materials)	Chair, balls, exercise (Bobath) ball, elastic bands (red, green, blue, black), cones/markers, objects for transfer tasks, dumbbells, wall bar or treatment table for support	Mat, dumbbells, cones/obstacles, wall bar or treatment table for support
4. What (procedures)	Reduced conventional program (all components retained, fewer sets/shorter duration) plus 18–20 min of turning practice: seated ball-reaching rotation; resisted trunk rotation; standing object transfer with rotation; figure-of-eight walking; trunk rotation in single-leg and tandem stance; sit-to-stand, 90° turn and return (Table 2)	Stretching, strengthening, balance, and gait training filling the whole session (Table 1)
5. Who provided	Four physiotherapists, each with at least 3 years of clinical experience, trained in both protocols before the study; each treated participants in both groups	Same four physiotherapists
6. How	Face to face, individually supervised	Face to face, individually supervised
7. Where	Physiotherapy and rehabilitation clinic, NRL Medical Center, Istanbul, Türkiye	Same
8. When and how much	3 sessions/week for 12 weeks (36 sessions), approximately 45–50 min per session, of which 18–20 min turning practice	3 sessions/week for 12 weeks (36 sessions), approximately 45–50 min per session
9. Tailoring	Elastic band resistance increased every 2 weeks (red, green, blue, black); black band reached in week 7 and maintained during weeks 9–12; difficulty adapted to safety and performance; rest between sets as needed	Repetitions, upper-limb support, base of support, reaching distance, and gait-task complexity progressed individually; dumbbell load increased every 2 weeks
10. Modifications	None	None
11. How well (planned)	Attendance recorded at each session; missed sessions rescheduled within the same week; minutes per component not recorded	Same
12. How well (actual)	All 25 participants attended all 36 sessions	All 25 participants attended all 36 sessions

Hoffmann TC, et al. Better reporting of interventions: template for intervention description and replication (TIDieR) checklist and guide. BMJ 2014;348:g1687.
